# A peek behind the curtain

**DOI:** 10.1007/s12471-018-1138-9

**Published:** 2018-08-02

**Authors:** M. Kamali-Sadeghian, P. T. G. Bot, R. Tukkie, H. J. Wellens, D. J. van Doorn

**Affiliations:** 1 Department of Cardiology, Spaarne Gasthuis, Haarlem, The Netherlands; 2 Department of Cardiology, Maastricht University Medical Center, Maastricht, The Netherlands

## Answer

**Fig. 1:**
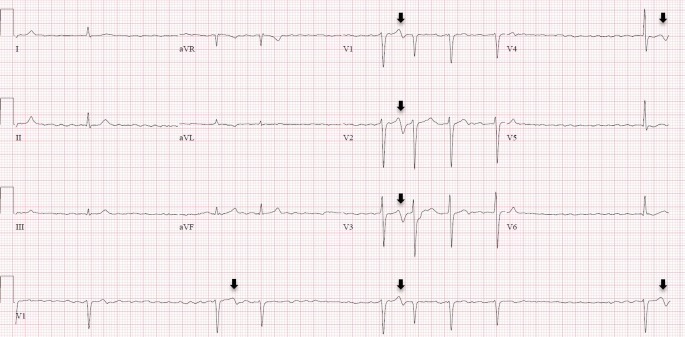
The arrows indicate the biphasic T waves in the precordial leads appearing after a preceding delay

The electrocardiogram (ECG) on admission shows atrial fibrillation with slow ventricular response of 42 beats/min. In some QRS complexes, prominent biphasic T waves are seen in the precordial leads V1–V4 (Fig. 1). This Wellens’ ECG sign is suggestive of critical proximal left anterior descending (LAD) stenosis [1]. The electrocardiographic features are characterised by either biphasic T waves or the more common deep T‑wave inversion in the anteroseptal leads. Furthermore, precordial ST-segment deviation, pathological Q waves and poor R‑wave progression should be absent. These ominous T‑wave inversions mostly occur in patients with a history of angina in a pain-free period, whereas angina can cause “pseudonormalisation” of the T waves [2].

Although the underlying mechanism remains elusive, it has been postulated that myocardial stunning due to oedema causes intramyocardial repolarisation inhomogeneity resulting in characteristic inversed or biphasic T waves [3]. Interestingly, the present ECG shows that the typical Wellens’ pattern only occurs after a long R‑R interval and thus a prolonged diastolic filling time, whereas rather short R‑R intervals are followed by normalised T waves. This phenomenon is presumably explained by the intermittent increase in left ventricular end-diastolic pressure impairing coronary perfusion and causing maximal ischaemia during contraction after a long R‑R interval with a large stroke volume.

**Fig. 2:**
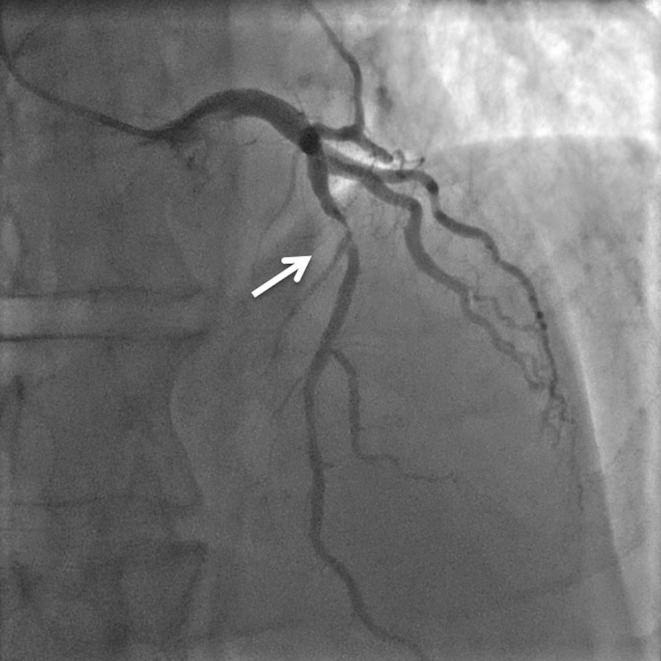
Coronary angiography revealing a subtotal stenosis in the proximal left anterior descending artery

In this patient, an emergent coronary angiogram indeed revealed a subtotal stenosis of the proximal LAD (Fig. 2). This lesion was successfully treated with the placement of a drug-eluting stent.

This case underlines that early recognition and urgent revascularisation is imperative in patients with Wellens’ syndrome, as delay in intervention may lead to anterior myocardial infarction [1].
